# A rare complication of people with inflammatory bowel disease after ileostomy: A case report

**DOI:** 10.1097/MD.0000000000035098

**Published:** 2023-09-15

**Authors:** Hong Jiang, Shengduo He, Huahong Wang

**Affiliations:** a Department of Gastroenterology, Peking University First Hospital, Beijing, China.

**Keywords:** anticoagulation, cavernous transformation of the portal vein, inflammatory bowel disease, prevention

## Abstract

**Rationale:**

Venous thromboembolism is one of the common complications of inflammatory bowel disease (IBD), which is a significant health problem worldwide. Factors such as inflammation, medications, and abdominal surgery, contribute to the increased risk of venous thromboembolism in patients with IBD. Cavernous transformation of the portal vein (CTPV) is a relatively rare complications of IBD. Subsequent portal hypertension could be fatal and the clinical treatment is difficult. Thus, early prevention is very crucial.

**Patient concerns:**

A 55-year-old man presented to our department with asthenia, hematemesis, and diarrhea. He was diagnosed with Crohn disease for 9 years. Two years ago, He suffered intestinal perforation and received enterectomy and ileostomy. And, anticoagulants were not given during perioperative period and after surgery because of the history of gastrointestinal bleeding.

**Diagnoses:**

The patient was given endoscopy inspection showing the varices of esophagus and gastric fundus near cardia. Contrast enhancement CT scan showed portal hypertension, CTPV, gastroesophageal varices, and splenomegaly. Then percutaneous transhepatic portography was performed to make a clear diagnosis.

**Interventions:**

Concerned about the risk of surgery, he refused surgical shunting. Conservative treatment was recommended due to technical difficulties instead of interventional therapy.

**Outcomes:**

And after a period of fasting, blood transfusion, and parenteral nutrition treatment, the patient did not experience any further vomiting or hematemesis.

**Lessons:**

Early identification and treatment of CTPV is difficult. Prevention such as early application of anticoagulant is necessary for patients with IBD undergoing surgery, particularly with simultaneous stoma formation. For IBD patients who are not suitable for anticoagulant therapy, postoperative follow-up monitoring should be more frequent and monitoring time should be extended.

## 1. Introduction

Venous thromboembolism (VTE) is one of the common complications of inflammatory bowel disease (IBD), which is a significant health problem worldwide. Factors such as inflammation, medications, and abdominal surgery, contribute to the increased risk of VTE in patients with IBD. Postoperative VTE events occur at increased rates in patients with IBD following colon and rectal surgery^[1]^ over the years, resulting in significantly increased cost and mortality.

Portal vein thrombosis (PVT) is known as a rare and poorly described complication of IBD. Cavernous transformation of the portal vein (CTPV), a sequela of extrahepatic and/or intrahepatic portal vein obstruction, is a relatively rare disease and its epidemiological data are limited by its infrequency. The related literature and information of CTPV is scanty specifically as one of the complications of IBD. It’s important to note that, though rare, outcomes of CTPV such as gut ischemia, development of portal hypertension and major bleeding can be fatal, which should be paid attention to.

## 2. Case report

A 55-year-old man presented to our department with asthenia, hematemesis, and diarrhea rather than abdominal pain. The patient had been diagnosed with Crohn disease (A3, L1, B2 + B3) for 9 years and hospitalized frequently due to intestinal obstruction. And he suffered intestinal perforation and received enterectomy and ileostomy in our hospital 2 years ago. Mesalazine was used before and after the surgery rather than glucocorticoids or biologics. The patient underwent ileostomy reversion in a stable stage of Crohn disease 10 months before this hospitalization and the surgery went well. However, after the second surgery, the patient suffered occasional abdominal pain and diarrhea with mesalazine as the maintenance therapy. And considering the patient’s history of gastrointestinal bleeding, anticoagulants were not given. It is worth noting that from the first surgery to this hospitalization, there is no glucocorticoids application.

**Figure 1.**
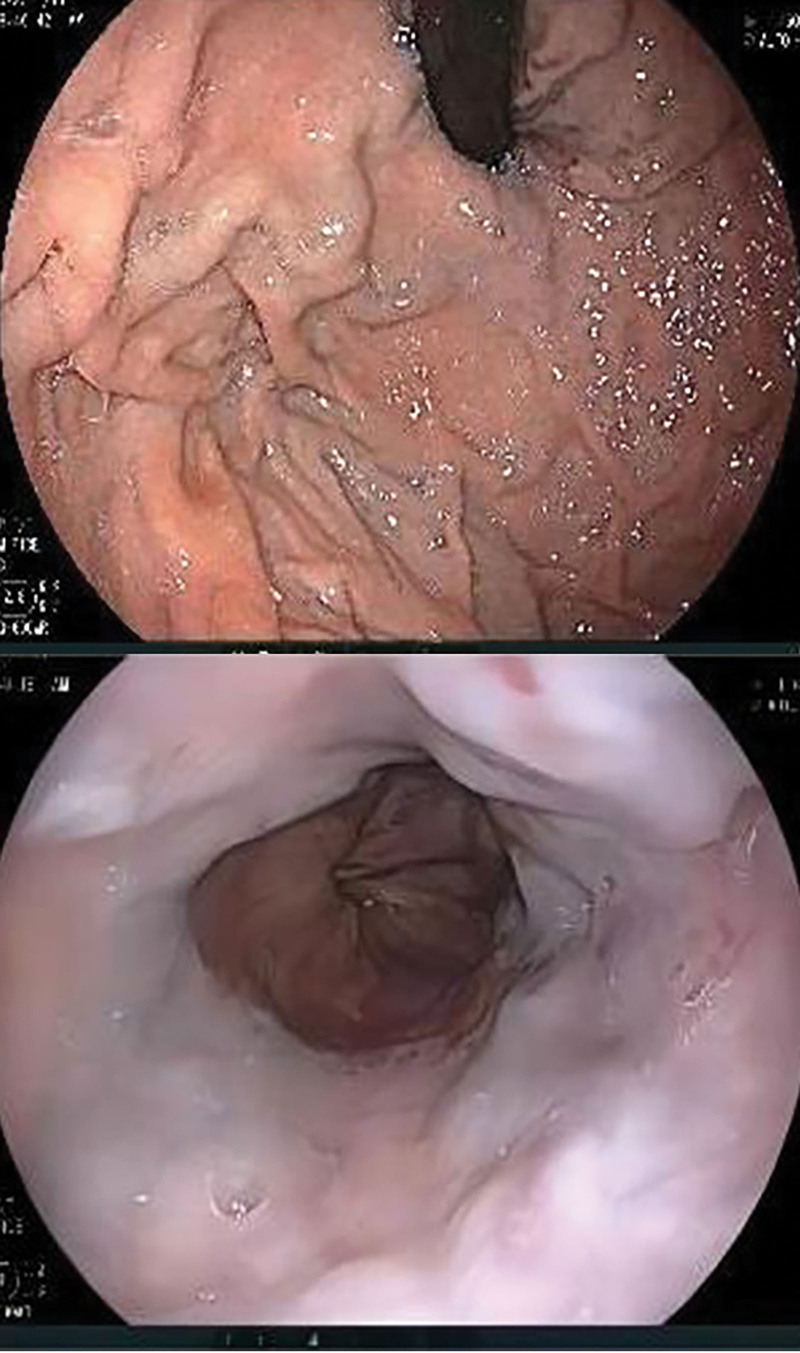
The gastroscopy showed esophageal varices.

**Figure 2.**
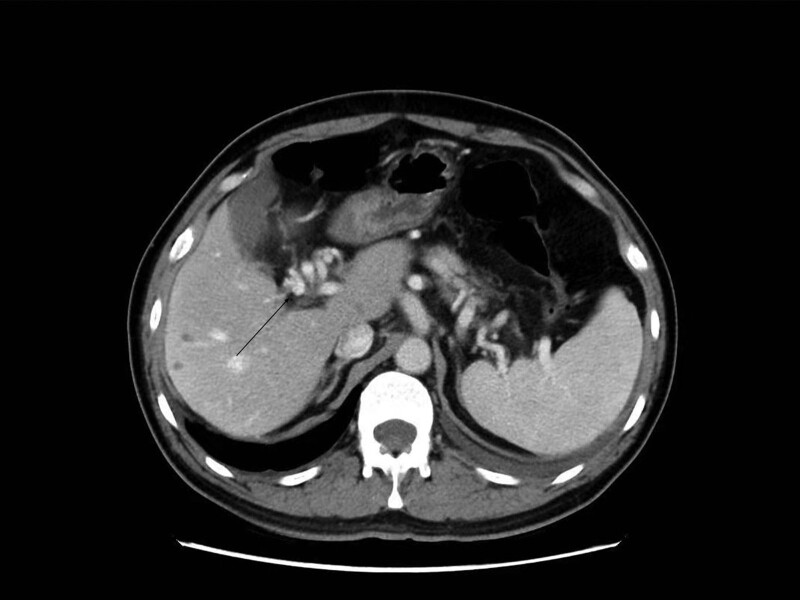
Axial CT scans showed numerous collateral vessels in the liver hilum (arrow).

**Figure 3.**
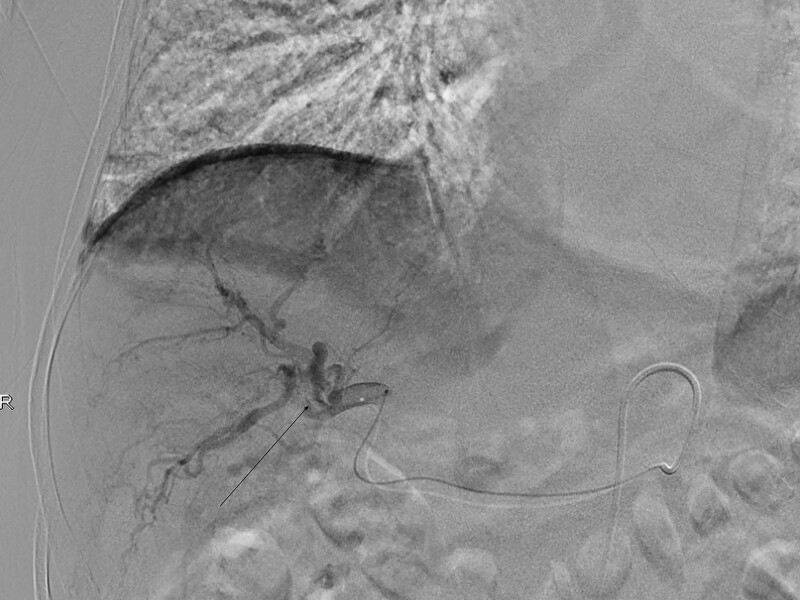
Percutaneous transhepatic portography (PTP) revealed numerous collateral vessels (arrow).

The patient was given endoscopy inspection showing the varices of esophagus and gastric fundus near cardia (Fig. 1). Hepatic hilar CTPV, as well as extrahepatic signs of portal hypertension (splenomegaly, gastroesophageal varices) were showed in contrast enhancement MRI and CT (Fig. 2), which were not found in previous inspections. Then percutaneous transhepatic portography (Fig. 3) was performed to make a clear diagnosis. Then the patients were examined to find out if there are other hidden diseases causing hypercoagulability. No abnormalities found during the screening of tumor, autoimmune disease, infectious disease, and thrombotic syndrome. A combination of relevant data, including his clinical presentation and examination, led to a diagnosis of hepatic hilar CTPV and consequent portal hypertension with gastrointestinal hemorrhage. The specified time of thrombosis event was not clear because the patient had no typical abdominal pain in the whole course of CTPV. Concerned about the risk of surgery, he refused surgical shunting. According to the results of percutaneous transhepatic portography, instead of modified transjugular intrahepatic portosystemic shunt and portal vein stenting, conservative treatment was recommended due to technical difficulties.

And after a period of fasting, blood transfusion and parenteral nutrition treatment, the patient did not experience any further vomiting or diarrhea. After the diet gradually returns to normal, the patient’s fatigue symptoms gradually alleviate. One year after discharged from hospital, the patient was followed up by telephone. He had visited several hospitals for interventional vascular surgery before coming to a conclusion that interventional vascular surgery was hard to perform and further interventional treatment is not recommended. He still had intermittent diarrhea but not with abdominal pain or bloody stool. However, He had not undergone colonoscopy for IBD assessment in the past year.

## 3. Discussion

It is a rare case report of IBD combined with CTPV. The patient diagnosed in Crohn disease, with a thorough evaluation revealing no evidence of inherited thrombophilia, experienced a major thrombotic event. Previous study^[2]^ showed colectomy and proctectomy with simultaneous stoma formation had significantly higher rates of VTE than those without. Surgery with simultaneous stoma formation might be a very important risk factor for the thrombotic event. Based on the description of the patient, occasional abdominal pain and diarrhea occurred after second surgery which indicated that active disease might contribute to the VTE. We therefore propose that regular monitoring and early application of antithrombotic drugs is necessary for IBD patient suffered surgery (especially for those who require a second surgery). For IBD patients who are not suitable for anticoagulant therapy, postoperative follow-up monitoring should be more frequent and monitoring time should be extended.

The general risk for VTE in patients with IBD is a 2- to 3-fold higher than that in patients without.^[3,4]^ The added insult of surgery puts IBD patients at even higher risk for a VTE event. For patients with IBD undergoing abdominal surgery, large population-based studies examined that the cumulative incidence of VTE within 12 months of hospital discharge was about1.2% to 1.6% for the surgical IBD cohort^[5,6]^ and many of VTE events occur after discharge.

CTPV is commonly secondary to PVT or portal vein obstruction. PVT is a relatively neglected complication of IBD, of which prevalence rates ranging from 0.17% to 1.7% of IBD patients.^[7]^Rates of secondary portal hypertensive complications were even lower. Initial presentations of PVT were generally nonspecific and the most common presenting complaint was abdominal pain. The most common abnormal findings of laboratory results were leukocytosis and elevated inflammatory markers. Fecal calprotectin levels were abnormal in some patients. As experienced by the patient, PVT was generally unexpected, the diagnosis of which was made on imaging (CT and MRI), highlighting the importance of periodic imaging examination in high-risk patients.

There is no special treatise on CTPV of people with IBD in past literatures, because of its rarity. The obstruction of intrahepatic portal vein resulted in the increase of portal pressure, which might precipitate the enlargement of the vessels peripheral to the main portal vein and promote the formation of periportal or intrahepatic venous collateral circulation, resulting in CTPV.^[8]^ When unexpectedly diagnosed with CTPV, the patient had already suffered portal hypertension, which added difficulty of treatment. As the patient experienced, CTPV could remain insidious for long-term presentation.

This unfortunate case serves as a reminder of the necessity of thromboprophylaxis in patients with IBD. International consensus guideline in 2021 recommends thromboprophylaxis is indicated during hospitalization of any cause in patients with IBD.^[9]^ Clear recommendations describing for how long patients with IBD after discharge should receive thromboprophylaxis is lacking. Rectal Surgeons (ASCRS) recommended continuing anticoagulation for 4 weeks after discharge for high-risk patients after abdominal surgery.^[10]^ Recent studies have demonstrated that the risk of VTE in patients with IBD remains elevated for 3 or even 6 months after hospital discharge.^[2,6]^For patients with IBD following colon and rectal surgery, extended prophylaxis could be considered. However, the side effects of gastrointestinal bleeding have baffled physicians and restrict application. Some other modifiable risk factors such as reducing preoperative anemia, malnutrition, and anesthesia time should also be paid attention.^[11]^

The limitation of the article is that this patient had refused surgery and no radiological intervention was indicated. Some researches^[7]^ regarded DOACs as the preferred anticoagulation for IBD associated PVT. However, the main portal vein of CTPV is usually considered to be unable to be recanalized with anticoagulation. Surgical and interventional treatment are the mainstay of treatment modality. And interventional techniques can benefit patients with CTPV by reducing the need for liver transplantation or increasing opportunities for liver transplantation.^[12]^

## 4. Conclusions

CTPV is a relatively rare disease and studies associated with IBD are limited. Early identification of CTPV is difficult and prevention is key. Early application of anticoagulant is necessary for patients with IBD undergoing surgery, particularly with simultaneous stoma formation. For IBD patients who are not suitable for anticoagulant therapy, postoperative follow-up monitoring should be more frequent and monitoring time should be extended.

## Author contributions

**Supervision:** Shengduo He, Huahong Wang.

**Writing – original draft:** Hong Jiang.

**Writing – review & editing:** Hong Jiang.
